# The prognostic implications of comorbidities in septic arthritis

**DOI:** 10.3389/fmed.2025.1566384

**Published:** 2025-07-29

**Authors:** Aiman Khudair, Ahmed Khudair, Alexandra E. Butler

**Affiliations:** ^1^School of Medicine, Royal College of Surgeons in Ireland - Medical University of Bahrain, Busaiteen, Bahrain; ^2^Research Department, Royal College of Surgeons in Ireland - Medical University of Bahrain, Busaiteen, Bahrain

**Keywords:** septic arthritis, comorbidities, diabetes mellitus, rheumatoid arthritis, chronic kidney disease, prognosis, mortality

## Abstract

Septic arthritis (SA) is an orthopedic emergency characterized by joint inflammation secondary to infectious etiologies, most commonly *Staphylococcus aureus*. The prompt recognition of SA is crucial due to its significant morbidity and mortality. Fever, along with a swollen, painful joint and limited range of motion, are typical manifestations; however, presentations can vary. The incidence of SA in adult populations is rising, accompanied by unfavorable mortality rates. This trend is further exacerbated by comorbid conditions that substantially influence outcomes. Among the literature, diabetes mellitus (DM), rheumatoid arthritis (RA), and chronic kidney disease (CKD) have emerged as key prognostic factors in SA. DM exacerbates the severity of SA through impairment of polymorphonuclear function, ultimately leading to increased susceptibility to infection and a higher risk of acquiring infection from atypical pathogens. CKD causes uremia-induced immune dysfunction leading to an immunocompromised state as well as repeated vascular access increasing infection susceptibility, leading to increased mortality. Patients with RA harbor an elevated risk of SA, attributed to immune dysregulation, immunosuppressive therapy, and diagnostic challenges. Additionally, these comorbidities can complicate the surgical management of SA and increase the likelihood of treatment failure. Therefore, given the rising burden of comorbid conditions worldwide and their impact on SA prognosis, healthcare professionals should remain vigilant when managing these factors. A holistic, multidisciplinary approach to management is vital to ensure that SA patients with these certain comorbid conditions experience fewer complications and improved survival. This mini-review aims to highlight the key comorbid conditions that impact the prognosis of SA patients.

## Introduction

Septic arthritis (SA), also known as infectious arthritis, is an inflammation of joint(s) secondary to an infectious etiology and is regarded as an orthopedic emergency (1, 2). In the United States, between 4 and 10 individuals per 100,000 are affected annually (2). Infectious agents are often bacterial, but can also involve viral, fungal, and mycobacterial pathogens (3). The most common organism to cause SA in roughly 37%–56% of cases is *Staphylococcus aureus* (4–6). SA typically presents as a single-joint infection; however, multiple joints can be affected in roughly 20% of cases, termed polyarticular septic arthritis (PASA), especially among patients who are immunocompromised or have severe sepsis, rheumatoid arthritis (RA), or multiple comorbid conditions (4, 7). PASA is further complicated by a higher mortality rate, higher treatment failure and diagnostic challenges due to the overlapping clinical features seen with other arthritic presentation such as RA (8–11).

In adults with SA, the knee joint is most often affected, comprising over 50% of cases whereas, in children, the hip joint is most affected. Other joints, such as the shoulder and elbow, are less frequently affected and can be more severe due to a delay in diagnosis (4, 7, 12, 13). This is more likely to occur in immunocompromised individuals, such as elderly patients, those with diabetes, intravenous drug users and those who are immunosuppressed.

Clinically, SA presents with an acute onset of fever and a warm, painful, swollen joint exhibiting a limited range of motion; however, presentation can vary between patients (7, 14). A systematic review involving 14 studies, revealed that in more than 50% of patients, the symptoms of joint pain, joint swelling, and fever occur with sensitivities of 85, 78, and 57% respectively (7, 15).

The gold standard used for diagnosis of SA involves bacterial isolation from the synovial fluid, with synovial culture being the most important test in all those with synovial fluid aspiration (7, 16). Additionally, real-time polymerase chain reaction (PCR) targeting the 16s rRNA gene has emerged as a potential modality in the diagnosis of bacterial SA. In comparison to results of synovial fluid culture, PCR harbors a sensitivity of 95% and a specificity of 97% (17). However, its definitive role in clinical performance is limited by its diagnostic performance, time to results, and inability to provide information on antibiotic sensitivity (18, 19). The management of SA is often aimed at reducing morbidity and mortality through prompt diagnosis and treatment, which often entails the use of broad-spectrum antibiotics until culture results are available, with organism identification to later narrow the choice of antibiotic (7, 14). Furthermore, joint drainage should be employed in the form of closed or open arthrotomy (3).

The rate of mortality associated with SA is significant; one national cohort study from Taiwan involving 31,491 patients found mortality rates of 4.3% at 30 days, 8.6% at 90 days, and 16.4% at 1 year (20). By comparison, a study from Denmark reported a 30-day mortality rate of 9.3% (21). Furthermore, in adult populations, the incidence of SA appears to be increasing and can be attributed to older age, increased use of immunosuppressants, underlying joint diseases and a rise in medical comorbidities (6, 22–25).

Certain prognostic factors, such as advanced age or the presence of certain comorbidities such as RA and diabetes mellitus (DM), significantly worsen outcomes. Therefore, the exploration of these factors is vital for the treatment and identification of SA (26–28).

A dramatic increase in 90-day mortality was found in one cohort study in patients who were 80 years and older (22%–69% compared to 7% in patients <80 years) (26, 28). Another cohort study identified a significant association between mortality and the presence of certain comorbidities such as DM, RA, bacteremia and low creatinine clearance, among others (27, 28). Furthermore, a systematic review of over 126 peer-reviewed studies in the adult population in community settings revealed a rise in multimorbidity, which is defined as having 2 or more chronic conditions simultaneously (29). The worldwide prevalence of multimorbidity is 37.2%, rising to a prevalence of 51% in those aged >60 (29, 30). Other systematic reviews based on adults in the community and in a healthcare-based setting report a prevalence of 42.4% (29, 31).

Therefore, these findings underscore the fact that comorbidities play a pivotal role in determining the prognosis of patients with SA. As the global burden of comorbid conditions continues to rise, the need to understand their underlying pathogenesis and impact on SA prognosis will only intensify. This mini-review therefore aims to highlight key comorbidities that play a significant role in the clinical trajectory of patients with SA, emphasizing the underlying pathogenesis and its clinical implications for patient care.

## Methods

### Search strategy

A literature search was conducted between December 2024 and January 2025 utilizing PubMed and Google Scholar. PubMed was used to retrieve peer-reviewed articles from indexed sources including MEDLINE. Google Scholar was also used to identify other relevant peer-reviewed journal articles from indexed databases such as ScienceDirect. The search included keywords such as “rheumatoid arthritis,” “chronic kidney disease,” “diabetes mellitus,” “mortality,” and “prognosis,” in relation to “septic arthritis.” Only articles written in the English language were considered. Articles were selected based on relevance, with no limitation on publication type or date.

### Diabetes mellitus

Diabetes mellitus is a chronic metabolic disorder characterized by hyperglycemia (32, 33). Type 1 DM results from the autoimmune destruction of pancreatic β cells, ultimately leading to insulin deficiency. By contrast, type 2 DM arises from the impaired ability of insulin-sensitive tissues to respond effectively to insulin, combined with impaired secretion of insulin by β cells in the pancreas (34–36). Given its global prevalence of approximately 537 million people, projected to rise to 783 million cases by 2045, it is an important disease to consider in terms of SA prognosis (37).

One study performed in Taiwan found that, among patients with *Staphylococcus aureus* SA, 41 (44.1%) had DM as the most common underlying disease (38). Of the five deaths reported, all occurred in immunocompromised patients with DM. Notably, DM was the only risk factor identified for mortality and was mentioned as the only significant poor prognostic factor (38). Similarly, a retrospective study in Qatar of patients aged 15 years or older with SA reported that DM was the most prevalent concomitant condition, present in 24 of the 56 patients (42.8%) (39). Another study in Paraguay investigating adult patients with SA reported that DM was again the most predominant comorbidity, affecting 63.6% of the 66 included cases (40).

One large prospective study determined that DM was an independent risk factor for the development of SA, among others (41). A retrospective study of 186 patients reported that DM carried 15.33 times higher odds of mortality compared to those without DM (42). However, another study involving 215 patients with SA concluded that DM was not a statistically significant risk factor for predicting 30-day mortality (21).

One mechanism through which DM can worsen the prognosis of SA is through its effect on the immune system by leading to an immunocompromised state. It is known that DM has a negative physiological impact on polymorphonuclear function, leading to increased susceptibility to infection (41). Notably, the rate of infection in those with type 2 DM was 47%–50% higher compared to the general population, with a positive correlation between infection rate and HbA1c (43–48). Previous studies have revealed that, in hyperglycemic conditions, neutrophil antibacterial activity, chemotaxis and adherence are impaired, while phagocytosis and bacterial killing mechanisms are also decreased (49–53). Attenuated neutrophil antibacterial activity can be explained by decreased ROS production, impaired superoxide generation, impaired neutrophil extracellular trap formation and degranulation, among others (54–58). Moreover, reduction in cytokine generation is also highlighted as a mechanism toward infection susceptibility, where decreased levels of type 1 interferon, interleukin (IL)-6, IL-2, and IL-10, among others, are impacted (58–60). Furthermore, monocyte respiratory burst activity was significantly reduced in individuals with blood glucose levels >11 mmol/L compared with those who had well-controlled DM or were healthy individuals (50, 61). Collectively, these processes lead DM patients to an immunocompromised state predisposing them to an increased infection risk. With regards to pathogen prevalence in DM patients, *Staphylococcus aureus* infection is typically more pronounced (21, 41). Furthermore, patients with DM are at increased risk for rarer, atypical organism infections in SA, including *Burkholderia pseudomallei*, *Candida albicans* and *Aspergillus* species, all of which can delay diagnosis and complicate treatment (62–68). DM predisposes patients to fungal infections through skin barrier integrity disruption and impaired antibody function (66, 69). Furthermore, microvascular and macrovascular complications in DM lead to a reduction of oxygen and blood supply, which negatively impacts the delivery of antibiotics and immune cells, amongst other factors (66, 69, 70). In addition, DM patients may be predisposed to rare joint involvement, such as the sternoclavicular joint, which is associated with complications and treatment challenges (71).

A study investigating 128 patients with acute SA undergoing surgical debridement identified that DM, among other risk factors, was an independent clinical predictor for single surgical debridement failure, with an odds ratio of 2.6 (72). However, conflicting evidence exists, where a recent systematic review of 30 studies comprising 8,586 native joint SA published in 2023 found that DM was not associated with surgical treatment failure (72–78). Importantly, patients with DM often require thorough preoperative evaluation and optimization to reduce perioperative morbidity and mortality. However, this crucial preparation may delay joint drainage, which may ultimately impact the prognosis (79).

**FIGURE 1:**
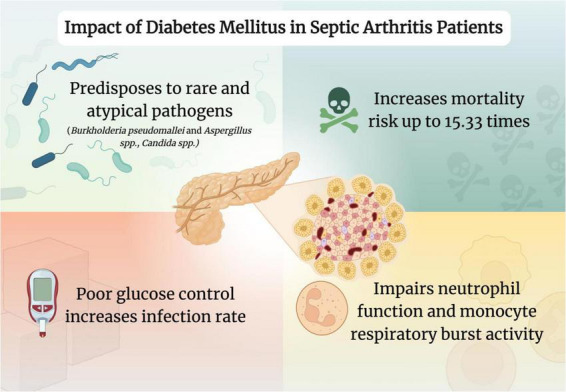
Impact of diabetes mellitus in septic arthritis patients. Created in www.biorender.com.

Although effective management of these patients is paramount, the evidence regarding DM’s role in treatment failure is conflicting. Nevertheless, DM has a substantial impact on prognosis and disease management (Figure 1). Therefore, healthcare professionals should utilize a multidisciplinary approach aiming for meticulous glycemic control and consistent monitoring to ensure the best possible outcomes for these patients.

### Chronic kidney disease

Chronic kidney disease (CKD) affects over 10% of the global population and is defined as decreased renal function as evidenced by a glomerular filtration rate of less than 60 mL/min per 1.73 m^2^ occurring for 3 months or more (80–82). In high-, middle-, and certain low-income countries, DM and hypertension are the primary causes of CKD (81, 83). Due to the progressive nature of CKD, patients ultimately require either dialysis or transplantation, both serving as forms of renal replacement therapy (82). In one study investigating end-stage renal disease (ESRD) patients, the incidence of SA was more than 50 times that of the general population (514.8 per 100,000 persons per year) (84). This finding was corroborated by other studies (85, 86). One proposed mechanism is uremia-induced immune dysfunction, which leads to a chronic immunocompromised state (84). In addition, the need for chronic vascular access increases susceptibility to infection (84, 87, 88).

Hematogenous spread, the commonest route of infection in SA occurs in over 70% of cases, thereby placing patients on hemodialysis at an increased risk (86, 89, 90). Furthermore, peritoneal dialysis may also be implicated (91). Due to frequent healthcare exposure, methicillin-resistant *Staphylococcus aureus* (MRSA) colonization is common among ESRD patients (92). One study determined that MRSA was implicated in 57.4% of SA cases in dialysis patients, with another reporting a rate of 58.3% in these patients (84, 93). The significance of this finding is immense as, in one study, those patients with native joint SA due to MRSA incurred a rate of treatment failure of 33.3% compared to those with methicillin-sensitive *Staphylococcus aureus* (MSSA) at 11.3% (94). Additionally, cases of MRSA SA were also associated with higher mortality, increased length of hospital stay and poorer clinical outcomes compared to MSSA SA (85, 86, 95–97). Moreover, common comorbid conditions in those with CKD, such as DM, further predispose to increased infection risk (98, 99).

In one study, hemodialysis-associated SA carried a 22% mortality rate, whereas another study reported a rate of only 7% (86, 100). The authors of the latter study owed these favorable outcomes to a multidisciplinary approach involving renal, orthopedic and infectious disease teams (86, 100). Therefore, this highlights the importance of coordinated care to improve prognosis in patients with ESRD with SA. Furthermore, dialysis access plays a pivotal role in prognosis. In a study by Yeh et al., the use of tunneled cuffed catheters was an independent predictor of positive blood cultures and in-hospital mortality (odds ratio 14.33) (93). Furthermore, elevated blood urea nitrogen (BUN) levels were associated with a higher likelihood of requiring a repeat washout procedure in one study (75). Additionally, chronic renal failure was strongly associated with mortality, with an odds ratio of 81.27 (42). Moreover, a Korean study that analyzed 89,120 hospitalizations for knee SA identified CKD as a risk factor for mortality (101).

Given the significant morbidity and mortality associated with SA in CKD patients, early recognition and a multidisciplinary approach are crucial for improving outcomes. Prioritizing infection prevention strategies, optimizing dialysis access and implementing prompt, focused management is essential toward improving the prognosis of SA in CKD patients.

### Inflammatory arthropathy

Inflammatory arthropathies are a group of joint diseases marked by joint inflammation, with RA being the most common (102–104). Spondyloarthropathies, including psoriatic arthritis, reactive arthritis and ankylosing spondylitis, are less common (103). RA is a chronic inflammatory disorder primarily involving the synovial joints that usually presents with swelling, pain, stiffness of joints, fever and malaise (105). Furthermore, it can also present with extra-articular manifestations involving the kidney, lung, heart, eye, skin, gastrointestinal and nervous system (106–109). RA predominantly affects females in a 3:1 ratio to males, with a prevalence of around 0.5%–1% in the adult population (110).

Of note, studies demonstrate that up to 40% of patients with SA have RA (12, 111). Additionally, in those with RA, the risk of SA, irrespective of therapy, increases by four- to fifteen-fold (21, 41, 112–114). Patients with seropositive RA treated with tumor necrosis factor (TNF) inhibitors, regardless of type, had a notable increased incidence of SA after the first year post-treatment commencement (115). This finding was also reported by another study which showed that the risk of SA doubled in RA patients treated with anti-TNF therapy (112). Another study confirmed that those taking disease-modifying antirheumatic drugs (DMARDs) compared to those without had a significantly increased risk of SA (114). A recent systematic literature review published in 2022 highlighted the increased risk for serious infection that the usage of biologic DMARDs carry in comparison to conventional synthetic DMARDs (116). Additionally, a recent narrative review also shared the same findings in regards to biologic DMARDs and encourages shared decision-making between healthcare providers and patients in regards to their own comorbidites and medication selection (117). According to the British Society for Rheumatology (BSR), they recommend that in those with an active infection, biological agents should not be started, while those carrying a heightened infection risk should utilize these agents with caution (118). Subsequently, the BSR recommends utilizing etanercept or adalimumab as first choice agents among patients with high infection risk (118).

This increased risk can be explained by decreased bactericidal activity within the synovial fluid and impaired phagocytosis due to the underlying disease and/or medications (12, 42, 119, 120). A proposed explanation for the increased SA risk in RA patients is the use of immunosuppressive medications, such as DMARDs and TNF inhibitors, which may predispose these patients to infection (42, 111, 112, 114, 121). In fact, individuals with RA are twice as likely to develop confirmed infection compared to those without the condition while matching for age and sex (111, 113). However, studies also show that patients who have not yet received steroids remain at a higher risk of infection, further alluding to the fact that underlying immune dysfunction along with the concomitant joint damage may be greater risk factors than the immunomodulatory drugs employed (121–123).

The treatment of SA in RA patients may also be impacted, as a study performed by Hunter et al., revealed that single surgical debridement failure had the highest odds ratio of 7.3 in those with a history of inflammatory arthropathy (72). However, a systematic review by Walinga et al. determined that the evidence regarding inflammatory arthritis being a positive risk factor for surgical treatment failure rate was conflicting (72, 73, 75, 124).

Besides the increased susceptibility to infection, diagnosing SA in RA patients can prove to ber challenging. A hot, inflamed joint could be mistaken for an RA flare-up rather than the development of SA, hence delaying the emergent diagnosis of SA (111, 114). A study by Favero et al., found that the time of onset on average was 25.07 ± 2.40 days in those with RA, compared to 14.30 ± 32.47 days in those without pre-existing joint disease (111). Another hindrance to early diagnosis is that RA patients often present late with normal white cell count and temperature (114). These factors all lead to a suboptimal prognosis. In terms of mortality prediction, one study concluded that RA was not a statistically significant predictor of 30-day mortality (21).

**FIGURE 2:**
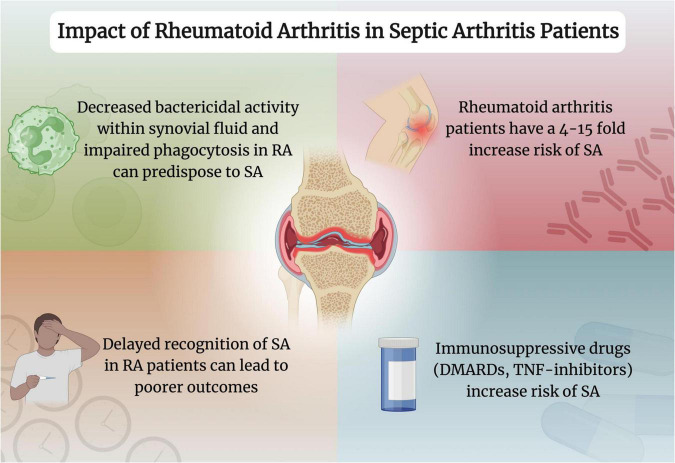
Impact of rheumatoid arthritis in septic arthritis patients. Created with www.biorender.com. RA, rheumatoid arthritis; SA, septic arthritis; DMARDs, disease-modifying antirheumatic drugs; TNF, tumor necrosis factor.

RA remains a critical factor in determining the prognosis in patients with SA (Figure 2). The prompt detection of SA in those with RA, alongside proper management, can ensure favorable outcomes in those with increased risk. Further evidence can help determine the best course of action to optimize the prognosis of SA in these patients.

### Cirrhosis

Cirrhosis, a chronic condition, is characterized by fibrosis and nodule formation in the liver as a result of chronic injury (125, 126). Some etiologies include non-alcoholic fatty liver disease, excessive alcohol consumption, hepatitis B or C infection, autoimmune and cholestatic diseases (127). Clinically, patients with cirrhosis may present with jaundice, ascites, hepatomegaly, spider nevi or encephalopathy (126). A study conducted in Taiwan aimed to analyze the occurrence of native SA in non-cirrhotic and cirrhotic patients, revealing that cirrhotic patients possessed a significantly higher risk of developing native SA compared to non-cirrhotic patients (128). Furthermore, patients with complicated cirrhosis (defined as those with refractory ascites, episodes of esophageal/gastric variceal bleeding, or hepatic encephalopathy episodes) were more susceptible to developing native SA compared to the patients with non-complicated cirrhosis (128). This may be attributed to the impaired phagocytic function of neutrophils and the innate immune system (128, 129). As such, cirrhosis represents an important comorbidity that can significantly affect the prognosis of SA and should be diligently considered in its management.

### Limitations

One limitation of our review is the relatively short search window conducted between December 2024 and January 2025. This narrow time frame could have potentially excluded newly published studies.

## Conclusion

Septic arthritis, an inflammation of one or more joints secondary to an infectious cause, remains a critical orthopedic emergency, particularly plaguing those with certain comorbidities. Studies indicate that CKD, DM and RA not only increase the risk of acquiring SA but also lead to poorer clinical prognosis with increased mortality or failure of treatment. The exploration of the complex interplay between these comorbidities and SA allows a deeper understanding for healthcare professionals to refine management strategies. Proactive assessment and management of these comorbidities is critical to minimize their negative implications on SA prognosis. A multidisciplinary approach involving a wide range of specialties including, but not limited to rheumatologists, nephrologists, orthopedics and infectious disease specialists, alongside prompt detection and timely treatment is imperative for an optimal patient prognosis. Further research is needed to determine the effective therapeutic regimens that are optimal in managing the comorbid conditions and preventing both the onset and progression of SA.
